# Optic chiasmatic potential by endoscopically implanted skull base microinvasive biosensor: a brain-machine interface approach for anterior visual pathway assessment

**DOI:** 10.7150/thno.71164

**Published:** 2022-04-11

**Authors:** Yikui Zhang, Shengjian Lu, Shenghai Huang, Zhonghao Yu, Tian Xia, Mengyun Li, Chen Yang, Yiyang Mao, Boyue Xu, Lixu Wang, Lei Xu, Jieliang Shi, Xingfang Zhu, Senmiao Zhu, Si Zhang, Haohua Qian, Yang Hu, Wei Li, Yunhai Tu, Wencan Wu

**Affiliations:** 1 The Eye Hospital, School of Ophthalmology & Optometry, Wenzhou Medical University; Wenzhou 325027, China.; 2 Medical Radiology Department, 2nd Affiliated Hospital, Wenzhou Medical University; Wenzhou 325027, China.; 3 Visual Function Core, National Eye Institute, National Institute of Health, NIH; Bethesda, United States.; 4 Department of Ophthalmology, Stanford University School of Medicine; Palo Alto, United States.; 5 Retinal Neurophysiology Section, National Eye Institute, National Institute of Health, NIH; Bethesda, United States.

**Keywords:** Microinvasive biosensor, trans-nasal endoscopy, skull base brain-machine interface, visual evoked potential, optic chiasmatic potential

## Abstract

**Background:** Visually evoked potential (VEP) is widely used to detect optic neuropathy in basic research and clinical practice. Traditionally, VEP is recorded non-invasively from the surface of the skull over the visual cortex. However, its trace amplitude is highly variable, largely due to intracranial modulation and artifacts. Therefore, a safe test with a strong and stable signal is highly desirable to assess optic nerve function, particularly in neurosurgical settings and animal experiments.

**Methods:** Minimally invasive trans-sphenoidal endoscopic recording of optic chiasmatic potential (OCP) was carried out with a titanium screw implanted onto the sphenoid bone beneath the optic chiasm in the goat, whose sphenoidal anatomy is more human-like than non-human primates.

**Results:** The implantation procedure was swift (within 30 min) and did not cause any detectable abnormality in fetching/moving behaviors, skull CT scans and ophthalmic tests after surgery. Compared with traditional VEP, the amplitude of OCP was 5-10 times stronger, more sensitive to weak light stimulus and its subtle changes, and was more repeatable, even under extremely low general anesthesia. Moreover, the OCP signal relied on ipsilateral light stimulation, and was abolished immediately after complete optic nerve (ON) transection. Through proof-of-concept experiments, we demonstrated several potential applications of the OCP device: (1) real-time detector of ON function, (2) detector of region-biased retinal sensitivity, and (3) therapeutic electrical stimulator for the optic nerve with low and thus safe excitation threshold.

**Conclusions:** OCP developed in this study will be valuable for both vision research and clinical practice. This study also provides a safe endoscopic approach to implant skull base brain-machine interface, and a feasible *in vivo* testbed (goat) for evaluating safety and efficacy of skull base brain-machine interface.

## Introduction

A visual signal is conveyed as trains of action potentials through the optic nerve, optic chiasma, optic tract, and optic radiation to the primary visual cortex in the occipital lobe, generally known as the visual pathway. Traditionally, the field potentials of the visual pathway can be recorded in two ways. Visual evoked potential (VEP) is recorded invasively on the surface of the visual cortex by an electrode penetrating the skull or non-invasively on the surface of the skull. Alternatively, optic nerve potential (ONP) is recorded from an electrode inserted into the retino-geniculo-occipital pathway by invasive open-skull surgery 1, 2. The signal of traditional VEP is low due to retino-genicular convergence and intracortical inhibition 3-5 and is variable due to artifacts caused by intra-cranial neural activities 6; whereas ONP recording is limited by its invasive procedure and restricted operating space 1, 7. A lack of a safe, sensitive and stable test with a strong signal to detect optic nerve integrity hampers the progress in the field of optic neuropathy.

**Figure 1:**
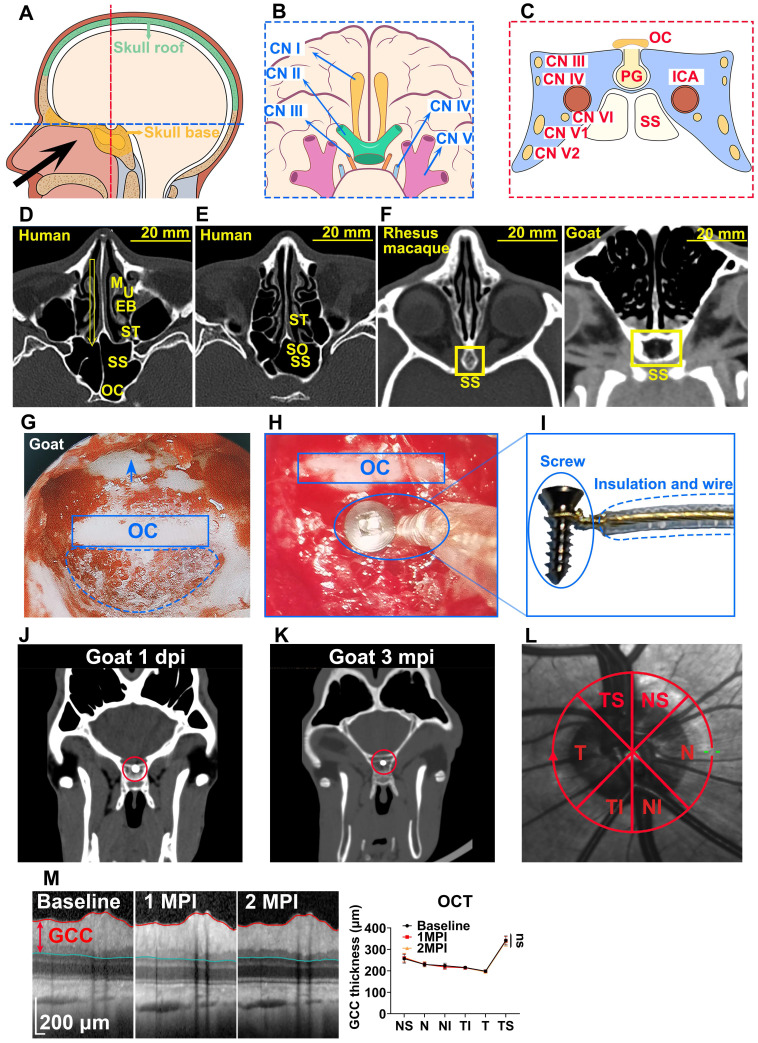
** Trans-nasal endoscopy is a feasible and safe approach to implant chiasmatic electrodes at the skull base. (A)** Human skull base (yellow shadow) and skull roof (green shadow). **(B, C)** Neurovascular tissues on the skull base on the horizontal and coronary planes along the blue and red lines are shown in (A). **(D, E)** CT scans of the nasal cavity and sphenoid sinus of human. Yellow arrow: trans-nasal route to skull base via sphenoid sinus. M: middle turbinate; U: uncinate process; EB: ethmoid bulla; ST: superior turbinate; SO: sphenoid ostium; SS: sphenoid sinus; OC: optic canal. **(F)** CT scan of the nasal cavity and sphenoid sinus of rhesus macaque and goat (yellow rectangle). **(G)** Endoscopic view of artificial airy SS in goat. The skull base is denoted by an arrow, the sphenoid bone body is indicated by a circle, and the optic canal is shown by a rectangle. **(H)** Endoscopic view of an implanted chiasmatic electrode (blue circle) beneath the optic canal. **(I)** Structure of self-made chiasmatic electrode consisting of a titanium screw, gold wire, and insulated outer layer. **(J, K)** Skull CT scans of the goat at 1 day and 3 months post chiasmatic electrode (circle) implantation. **(L)** Demonstration of retinal OCT circle scan around the optic nerve head (S: superior; I: inferior;T: temple, N: nasal), the GCC thickness of six regions was measured. **(M)** Representative OCT images and quantification of GCC thickness before and 1, 2 months after implantation, n = 6 goats, two-way ANOVA with Dunnett's multiple comparisons (compared with baseline), data are presented as mean ± s.e.m, ns: not significant.

The aim is to achieve simultaneous large-amplitude high-repeatability ONP signal recording while not requiring the invasive open-skull procedure. Minimally invasive trans-sphenoidal endoscopic surgery is an approach to expose the skull base, where optic canal and chiasm are located, without the need for open-skull surgery (**Figure 1A**) 8-10. Furthermore, the endoscopic approach provides a magnified view under bright illumination, enabling surgeons to treat skull base disorders with much fewer complications than conventional open-skull surgery 9, 10. To explore the possibility of trans-sphenoidal endoscopic implantation of skull base electrode, we chose goat as an animal model, whose sphenoidal anatomy and size are similar to that of humans 11, 12. After creating an artificial sphenoid sinus, we successfully accessed the optic chiasm and implanted an electrode into the sphenoid bone beneath the optic chiasm.

The implantation procedure was swift (within 30 min) and safe. We did not find any detectable deficit in vision or behavior over months. Notably, a flash optic chiasmatic potential (f-OCP), detected from the chiasmatic electrode, was 5-10 times stronger, more sensitive, and repeatable than traditional flash visual evoked potential (f-VEP). Mechanistically, we explored the origin of OCP and confirmed that its signal relied on ipsilateral light stimulation and the integrity of the optic nerve. Using the same OCP device, we further demonstrated several potential applications in evaluating and treating optic neuropathy.

Our work will be valuable for animal model studies used for visual function and outcome measurement of treatments and applicable to clinical cases with traumatic visual defects. In addition, we provided a new route for skull base brain-machine interface (BMI) with minimal surgical invasion, which can potentially monitor and manipulate neural activities of the frontal cortex and cranial nerves (CN) I to VI, the blood flow of the intra-carotid artery (ICA) and cavernous sinus, and endocrine activities of the pituitary gland (**Figure 1B, C**).

## Results

### Trans-sphenoidal endoscopy as a feasible and safe approach for implanting a chiasmatic electrode at the skull base

The sphenoid sinus (SS) provides an important entrance to the skull base, which can be accessed trans-nasally after partial removal of the ethmoid bulla (EB) and superior turbinate (ST) via minimally invasive endoscopy (Figure 1D). Furthermore, the diameter of SS is around 20 mm 13, offering a large operating space for the implantation of relatively large and complex BMIs (Figure 1D). Small BMIs at the skull base can enter SS non-invasively through the sphenoid ostium (SO), a 3 mm wide natural opening of SS 14 (Figure 1E).

We employed goats to test the feasibility and safety of trans-sphenoidal implantation of the skull base electrode, as the sphenoidal anatomy and size of the goat and sheep are similar to that of humans 11, 12, even more so than monkeys 15 (Figure 1F). Since airy SS does not exist in goats (Figure 1F), we created an artificial airy SS using clinical settings of trans-nasal endoscopy, the size of which (approximately 14 mm in width) was comparable to that of human SS (approximately 20 mm) (Figure 1F). After exposing the optic chiasm located on the posterior wall of the artificial SS (Figure 1G), we implanted a custom-made electrode into the sphenoid bone beneath the optic chiasm (Figure 1H). The electrode consisted of a titanium bone screw (1.5 mm in diameter, 5 mm in length) and an insulated golden wire fastened to the screw (Figure 1I) capable of recording and stimulating electrical signals. The whole implantation surgery from skin incision to skin closure took 0.5 h or less.

Trans-sphenoidal implantation of the chiasmatic electrode was safe. The goats were able to walk and fetch food normally within 30 min after recovery from general anesthesia and continued to behave normally over 3 months postoperatively (**Movie S1**). Additionally, skull CT scans at 1 day and 3 months postoperatively did not find any cranial, orbital or nasal abnormality such as intra-cranial infection or hemorrhage (**Figure 1J-K, Movie S2-3**). Furthermore, the electrode implanted beneath the optic chiasm did not cause any visual abnormality in the following *in vivo* tests. Retinal optic coherent tomography (OCT) taken at baseline before surgery, 1, 2 months post-implantation (mpi) did not reveal any change in the thickness of the ganglion cell complex (GCC) (**Figure 1L-M**). Consistently, the amplitude of pattern electroretinogram (p-ERG), which reflected the function of the retinal ganglion cells (RGCs), did not show a significant reduction over 2 mpi (**Figure S1A-B**).

### Higher amplitude, larger dynamic range, and higher sensitivity of f-OCP than f-VEP

**Figure 2:**
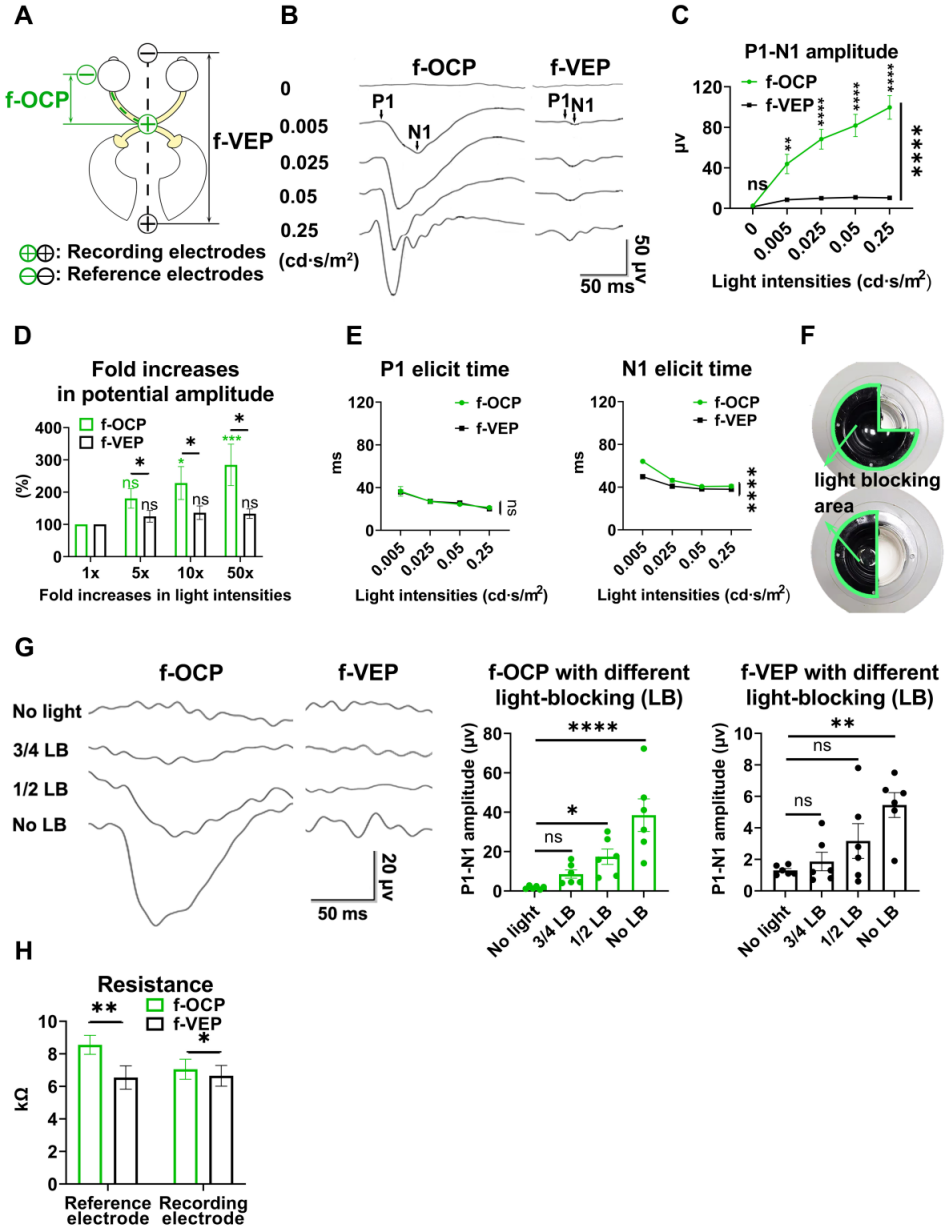
** F-OCP is of higher amplitude, larger dynamic range, and higher sensitivity than f-VEP. (A)** Scheme of electrodes placement of f-OCP and f-VEP. **(B)** Representative waveforms of f-OCP and f-VEP at different light intensities. Amplitude was measured from N1 to P1. **(C)** Comparison of P1-N1 amplitudes between f-OCP and f-VEP at different light intensities, n = 4 goats (no light stimulation), n = 7 goats (other light intensities), two-way ANOVA with Šídák's multiple comparisons, p < 0.0001 between f-OCP and f-VEP. **(D)** Comparison of fold increases in potential amplitude between f-OCP and f-VEP at different fold increases of light intensities, n = 7 goats, two-way ANOVA with Tukey's multiple comparisons (f-OCP vs f-VEP) and Friedman test Dunn's multiple comparisons (5x, 10x, and 50x compared with 1x). **(E)** Quantification of implicit times of P1 and N1 between f-OCP and f-VEP, n = 4 goats, two-way ANOVA, p = 0.9377 (P1 implicit time), p < 0.0001 (N1 implicit time). **(F)** Images of light blockers used to patch three or two quadrants of light-emitting areas of the mini-Ganzfeld stimulator. **(G)** Representative waveforms (left panel) and P1-N1 amplitudes of f-OCP and f-VEP (right panel) with or without light blocking (LB), n = 6 goats, one-way ANOVA with Dunnett's multiple comparisons. **(H)** Comparison of recording and reference electrodes resistance between f-OCP and f-VEP, n = 5 goats, paired t-test, p = 0.0011 (Reference), p = 0.0446 (Recording). Data are presented as mean ± s.e.m, ns: not significant, *: p < 0.05, **: p < 0.01, ***: p < 0.001, ****: p < 0.0001.

We compared f-OCP signals using the chiasmatic electrode and traditional f-VEP at the occipital bone by recording them simultaneously (Figure 2A). Intriguingly, the amplitudes of f-OCP at different light intensities (0.005-0.25 cd·s/m^2^) were approximately 5-10 times larger than those of f-VEP (Figure 2B-C). Furthermore, f-OCP amplitude increased as a function of light intensity ranging from 0.005 to 0.25 cd·s/m^2^ (Figure 2D). On the contrary, the amplitude of f-VEP was saturated at light intensity beyond 0.05 cd·s/m^2^ (Figure 2D), presumably due to the retina-geniculate convergence 3, 4 and the inhibitory processes/compensatory mechanism within the cortical lamina 5, 16. Consistently, the amplitude of f-OCP showed a more significant increase than that of f-VEP in response to higher light intensity (Figure 2D), indicating that f-OCP had a larger dynamic range and was more sensitive to small changes. There was no significant difference in the implicit times of P1 between f-OCP and f-VEP. The implicit time of N1 in f-OCP was slightly larger than f-VEP, especially at 0.005 cd·s/m^2^ (Figure 2E).

F-OCP was also more sensitive in detecting weak light stimulus indetectable by f-VEP. As shown in **Figure 2F**, we weakened light stimulation using a light blocker to partially cover the mini-Ganzfeld stimulator. When three-quarters of the stimulator was blocked at the light intensity of 0.015 cd·s/m^2^, neither f-VEP nor f-OCP was elicited,and when half of the stimulator was blocked, a typical f-OCP with a large negative deflection was elicited, whereas f-VEP was still indistinguishable from the background. Both f-OCP and f-VEP were elicited in the absence of any blockage (**Figure 2G**).

We measured the electrical impedance of the recording and reference electrodes of f-OCP and f-VEP simultaneously to exclude the effect of electrical resistance on amplitude, and found that the electrical resistance of the f-OCP reference electrode was slightly but significantly higher than f-VEP (**Figure 2H**). That might be because the f-OCP reference electrode was inserted into the subcutaneous space rather than directly connected to a skull screw as the f-VEP reference electrode.

These results indicated that compared with f-VEP, the amplitude of f-OCP was more sensitive to light stimulation and its subtle changes, making it potentially a better tool in monitoring dynamic changes of ON function during ON degeneration and regeneration.

### Higher repeatability of OCP than VEP

**Figure 3:**
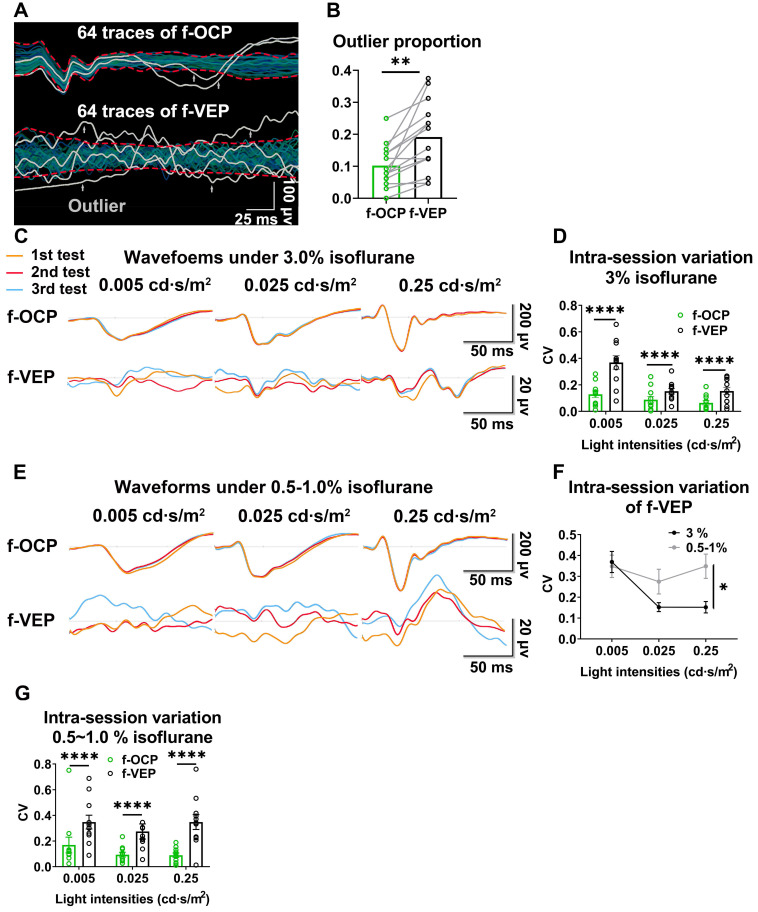
** OCP is more reproducible than VEP. (A)** Representative 64 traces of f-OCP and f-VEP in a single test. “Outlier” traces are labeled in white, and the contour of group traces are labeled as red dash lines. **(B)** Comparison of outlier proportion between f-OCP and f-VEP, n = 14 goats, paired t-test, p = 0.0013. **(C)** Representative waveforms of f-OCP and f-VEP recorded in three consecutive tests in the same session under anesthesia with 3.0% isoflurane. **(D)** Quantification of (C) intra-session CV of P1-N1 amplitude, n = 11 goats, two-way ANOVA with Tukey's multiple comparisons. **(E)** Representative waveforms of f-OCP and f-VEP were recorded in three consecutive tests in the same session under anesthesia with 0.5-1.0% isoflurane. **(F)** Comparison of the intra-session CV of f-VEP P1-N1 amplitudes under 0.5-1.0 and 3.0% isoflurane anesthesia, n = 11 goats, two-way ANOVA. **(G)** Comparison of the intra-session CV of P1-N1 amplitude between f-OCP and f-VEP with 0.5-1.0% isoflurane anesthesia, n = 11 goats, two-way ANOVA with Tukey's multiple comparisons. Data were presented as mean ± s.e.m. *: p < 0.05, **: p < 0.01, ****: p < 0.0001.

In the same anesthetic session, 64 consecutive traces of both f-VEP and f-OCP were simultaneously recorded for each light intensity, and then averaged to yield one waveform. As shown in **Figures 3A and B**, compared with f-VEP, f-OCP had significantly fewer “outlier traces”, which strayed away from the contour formed by group traces. Additionally, waveforms of f-OCP at three consecutive tests within the same anesthetic session were more repeatable than those of f-VEP (**Figure 3C**), which was confirmed by the quantification of the intra-session coefficient of variation (CV) in the amplitudes of f-OCP and f-VEP (**Figure 3D**).

Since the retino-geniculo-cortical pathway across the brain modulated f-VEP, its instability might originate from variable intra-cranial neuronal activities, which could be suppressed by isoflurane, a commonly used anesthetic, in a dose-dependent manner 17, 18. In contrast, f-OCP was less influenced by intra-cranial neuronal activities since it was recorded from ON extra-cranially. We tested our hypothesis by lowering the isoflurane concentration from 3.0% to 0.5-1.0% (the lowest concentration to keep goat under very light general anesthesia) and found that the f-VEP waveforms became more variable among three consecutive tests (**Figure 3E**) with significantly larger amplitude CVs (**Figure 3F**). On the contrary, the f-OCP waveforms, which were recorded simultaneously with f-VEP, remained repeatable despite the lower concentration of isoflurane (**Figure 3C, E**), and the amplitude CVs of f-OCP at different light intensities were still much lower than those of f-VEP (**Figure 3G**). These data suggested that f-OCP was more repeatable than f-VEP, probably because it was directly recorded from the ON and thus not influenced by intra-cranial neuronal activities.

### F-OCP reliance ipsilateral light stimulation and ON

**Figure 4:**
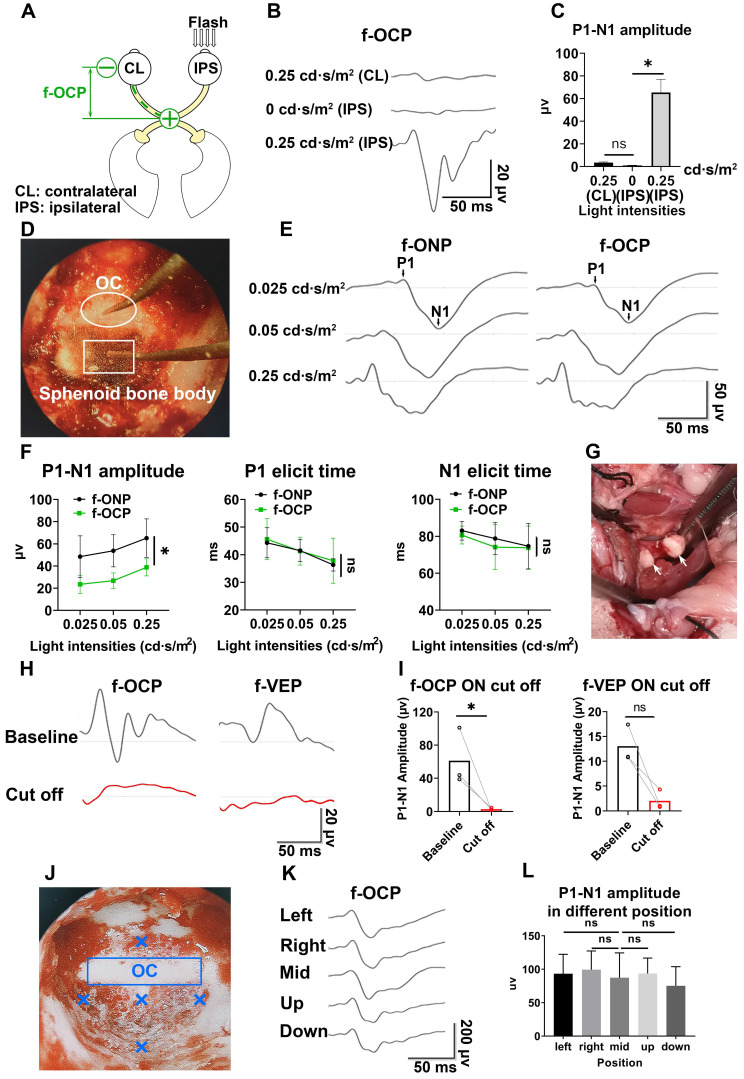
** F-OCP relies on ipsilateral visual stimulation and ON. (A)** Schematic of stimulating ipsilateral eye (IPS) and recording contralateral eye (CL). **(B, C)** Waveforms and quantification of f-OCP under 0, 0.25 cd·s/m^2^ (IPS) and 0.25 cd·s/m^2^ (CL). **(D)** Endoscopic image showing the simultaneous recording of f-OCP (the recording electrode is shown in a box) and f-ONP (the recording electrode is shown in a circle). **(E)** Representative waveforms of f-OCP and f-ONP under different light intensities. **(F)** Comparisons of P1-N1 amplitude (left panel), P1 elicit time (mid panel), and N1 elicit time (right panel) between f-OCP and f-ONP, n = 3 goats, two-way ANOVA, data are presented as mean ± s.e.m. **(G)** Image showing retrobulbar ON cut off; the cutoff ends are labeled by white arrows. **(H, I)** Representative waveforms and quantification of f-OCP and f-VEP amplitudes before and after ON cutoff. n = 3 goats. Ratio paired t-test for f-OCP ON cutoff, p = 0.0341. Wilcoxon test for f-VEP ON cutoff, p = 0.1000. **(J)** Endoscopic image showing five different locations of the recording electrode. **(K, L)** Representative waveforms of f-OCP and comparisons of P1-N1 amplitudes when the recording electrode was placed at different locations. n = 3 goats, one-way ANOVA with Dunnett's multiple comparisons (compared with mid). Data are presented as mean ± s.e.m, ns: not significant, *: p < 0.05.

In the absence of light stimulus, the f-OCP signal disappeared (**Figure 2B-C**), indicating the signal was elicited by visual stimulation. Additionally, light stimulation of the ipsilateral eye did not elicit f-OCP on the contralateral ON (**Figure 4A-C**). To explore the origin of f-OCP, we inserted a recording electrode into the optic chiasm to record chiasmatic ONP (f-ONP) (**Figure 4D**). The waveforms of f-OCP and f-ONP appeared similar (**Figure 4E**), although the amplitude of the former was significantly smaller (**Figure 4F**). The elicit times of either N1 or P1 were statistically the same between f-OCP and f-ONP (**Figure 4F**). A previous study also found that ONP was more clearly recorded on the ON than on the surrounding tissues 7.

Next, when we transected the retrobulbar ON and performed complete hemostasis (**Figure 4G**), both f-OCP and f-VEP signals vanished (**Figure 4H-I**). On the contrary, the flash electroretinogram (f-ERG) and p-ERG waveforms still existed following ON cutoff; however, their amplitudes reduced significantly (**Figure S2A-B**).

It is of note that when the ON was cut off without hemostasis, both f-OCP and f-VEP were still elicitable, although their amplitudes decreased (**Figure S2C-D**). We hypothesized that blood electrolytes might convey electrical signals between the ON cutoff ends. This hypothesis was supported by the following observation (**Figure S2E-G**): when we cut off the ON and carried out complete hemostasis using hemostatic powder, f-OCP disappeared (1^st^ test); a few minutes later, f-OCP re-appeared following visible bleeding (2^nd^ test); when we performed hemostasis using hemostatic powder again, f-OCP re-disappeared (3^rd^ test). F-ERG, taken immediately after the 3^rd^ f-OCP, showed regular a and b waves. When we placed the recording electrode at different locations of the skull base, the f-OCP waveform and amplitude did not change significantly (**Figure 4J-L**), indicating f-OCP was a far-field potential 1, 19, 20.

### Broad applications of the chiasmatic electrode in monitoring and treating optic neuropathy

#### Real-time detector of ON function

**Figure 5:**
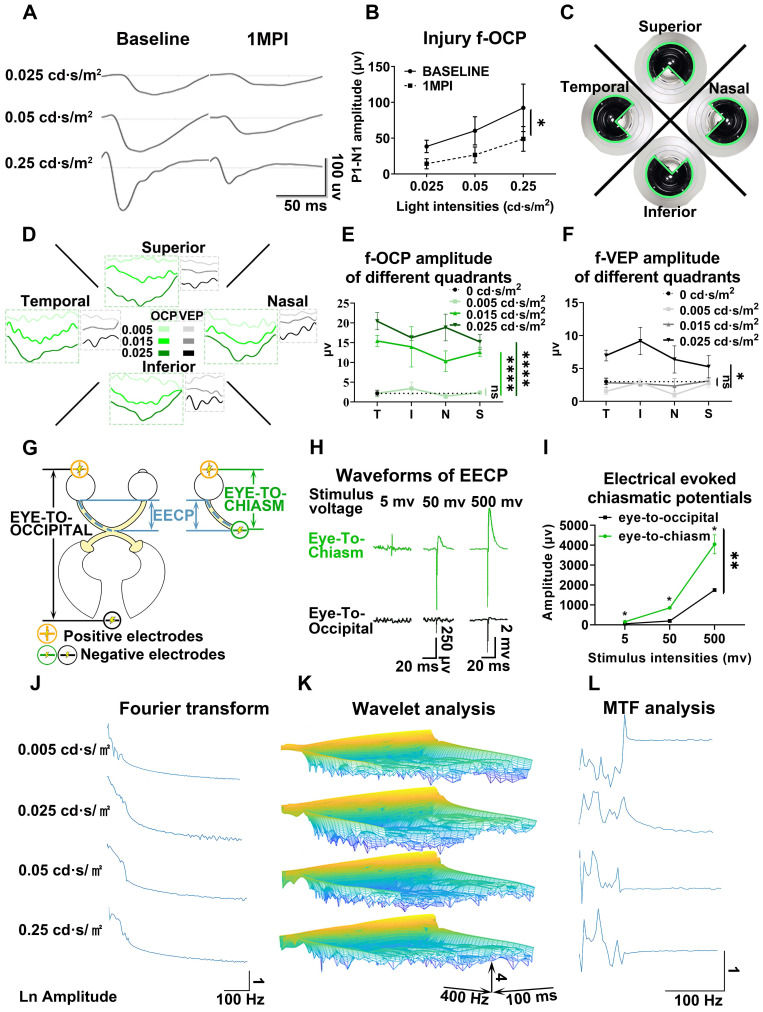
** Chiasmatic electrode has broad potential applications in monitoring and treating optic neuropathy. (A)** Representative waveforms of f-OCP before and 1 month after retrobulbar ON crush under different light intensities. **(B)** Quantification of f-OCP P1-N1 amplitude before and after crush, n = 3 goats, two-way ANOVA, p = 0.0324. **(C)** Demonstration of the custom-made region-biased retinal sensitivity detector, which allowed light emission from a quadrant. **(D)** Representative waveforms of f-OCP and f-VEP in response to light stimulation from a quadrant. **(E, F)** Quantification of (D). n = 3 goats, two-way ANOVA with Dunnett's multiple comparisons (compared with 0 cd·s/m^2^). **(G)** Schematic of two electrical fields established with either the chiasmatic skull base electrode (eye-to-chiasm field) or a conventional occipital electrode (eye-to-occipital field). **(H)** Representative waveforms of electrically evoked potentials between the eye and optic chiasm under two different electrical fields in (G). **(I)** Quantification of (H). n = 3 goats, two-way ANOVA with Tukey's multiple comparisons. **(J, K, L)** Fourier transform, wavelet and MTF analyses of f-OCP under different light intensities. Data are presented as mean ± s.e.m, ns: not significant, *: p < 0.05, **: p < 0.01, ****: p < 0.0001.

As shown in **Figures 5A and B**, the amplitude of f-OCP reduced significantly at 1-month post-injury (mpi) after retrobulbar ON crush. Compared with f-VEP, f-OCP had a higher signal, was more sensitive, and remained repeatable under very light general anesthesia. Additionally, the chiasmatic electrode remained fastened to the sphenoid bone and did not cause detectable abnormality. These features endow the chiasmatic electrode with the potential to be a real-time wireless detector of ON function without the need for anesthesia.

#### Detector of region-biased retinal sensitivity

Perimeter is widely used in clinical practice to detect visual field deficits which can caused by regional retinal dysfunction. Traditional perimeter test needs patient cooperation and thus cannot be carried out in unconscious patients or animal studies. Multi-focal VEP can detect visual field deficits, however, its application was limited by low signal-to-noise ratio, complicated software, and expensive hardware 21, 22. As shown in **Figure 5C**, we used a custom-made light blocker to cover three quadrants of the mini-ganzfeld stimulator, leaving one quadrant exposed (superior, nasal, temporal, or inferior quadrant). We recorded f-OCP and f-VEP simultaneously in response to increased light stimulation for each exposed quadrant. At a light intensity of 0.005 cd·s/m^2^, there were no detectable f-OCP or f-VEP. When the light intensity increased to 0.015 cd·s/m^2^, f-OCP could be elicited by light stimulation from each quadrant, whereas f-VEP was still indistinguishable from the background. At the light intensity of 0.025 cd·s/m^2^, both f-OCP and f-VEP were elicited (**Figure 5D-F**). Our work forms the framework of region-biased retinal sensitivity detector using f-OCP to measure light sensitivity threshold. Future design of more spatially defined light stimuli is warranted for more precise retinotopic sensitivity mapping.

#### Safe therapeutic electrical stimulator with low excitation threshold

The electrical field has been proven to promote guided axonal regeneration 23-25. However, its clinical application was hampered by safety issues. Here, we showed that the same electrical stimulation level applied via the chiasmatic electrode resulted in much higher electrically evoked potential between eye and optic chiasm than conventional occipital electrode (**Figure 5G-I**). Therefore, a therapeutic electrical field can be established by the chiasmatic BMI with much lower and thus safer electrical stimulation.

## Materials and Methods

### Animals

Animal experiments were conducted following the Association for Research in Vision and Ophthalmology (ARVO) Statement for the Use of Animals in Ophthalmic and Vision Research guidelines. Experimental protocols were approved by the Institutional Animal Care and Use Committee in the Wenzhou Medical University (Wenzhou, China, ID number: wydw2020-0789). Male Saanen goats at the age of 4-7 months, weighing 19-22 kg were purchased from the Caimu Livestock Company (Hangzhou, China) and housed in the animal facility of the Wenzhou Medical University under a normal light/dark cycle (12/12 h) with food *ad libitum*. Room temperature was maintained at 21 ± 2 °C.

### Study Design

One surgeon performed all surgeries, and the investigators collecting and analyzing the data were blinded to the grouping. No data outliers were excluded. Due to ethical issues and limitations of housing space and other resources, we used 2-14 goats in each test according to our previous experimental experience and preliminary study results.

### Trans-sphenoidal endoscopic implantation of skull base electrode in goats

Goats were anesthetized with 3% isoflurane at a rate of 3 L/min (RWD Life Science Co., Ltd, China). A skin incision on the nose was made, and the underlying nasal bone was removed to access the nasal cavity. The sphenoid bone was exposed using trans-nasal endoscopic devices (Delong, HD380B; Medtronic, Integrated Power Console ENT Surgery, 1898001; Medtronic, 1884004). An artificial sphenoid sinus was created using an endoscopic microdrill (Medtronic, diamond microdrill, 1882969) and an aspirator to expose the chiasmatic ON within the sphenoid bone. A custom-made skull base BMI, consisting of a titanium screw (Xianzhongbang Titanium Biomaterial Ltd., 1.5-5 mm, China), a golden wire (Jinghua Optoelectronics Technology Ltd., V-88, China), and an insulating outer layer (Shanghai Weicheng Electronics and electrical appliances Co., Ltd., 0.6 mm, China) (**Figure 1I**), was implanted trans-nasally and fastened into the sphenoid bony body beneath the optic chiasm using a screwdriver (**Figure 1H**). After implantation, the nasal cavity was irrigated with povidone-iodine, and the nasal skin incision was closed with 3-0 sutures. Subsequently, anesthesia recovery was conducted to awaken the goat.

### Retrobulbar ON crush/transection by lateral orbitotomy in goats

The detailed surgical procedure to expose the retrobulbar ON in goats was described previously 12. Briefly, after general anesthesia by xylazine (3 mg/kg, IM) or isoflurane (3% isoflurane, 3 L/min), lateral orbitotomy was performed. The retrobulbar ON was exposed after blunt dissection of the intra-orbital tissue and retraction of extra-ocular muscles. Then the ON was either crushed by a hemostat or transected incrementally by a sharp blade.

A hemostat was used to compress the distal ON before transection to achieve complete hemostasis after ON transection. During incremental transection, special attention was paid not to injure the ophthalmic artery or central retinal artery. For the first goat receiving ON transection, we performed fundus fluorescein angiography (FFA) 26 to confirm the integrity of retinal vascular filling (**Figure S3**). After transection, absorbable surgical hemostatic powder (SM0002, Arista™ AH, USA) was applied to the cutoff site to control bleeding.

### F-VEP, f-OCP, and f-ONP recording

F-VEP recording in goats has been described previously 12. Briefly, goats were anesthetized by isoflurane. The recording and reference electrodes were placed at the occipital bone and the frontal bone, respectively, using skull screws; the ground needle electrode was inserted into the subcutaneous space beneath the reference electrode. After adapting to the testing environment for 5 min, the f-VEP was consecutively recorded at different light intensities. For each intensity, consecutive 64 traces in response to flash stimulation were averaged to yield one waveform. The first positive peak in the f-VEP waveform was designated as P1, the first negative peak as N1. The P1-N1 amplitudes were measured from N1 to P1.

F-OCP was recorded simultaneously with f-VEP with their electrodes connected to a two-channel amplifier (GT-2008V-III, GOTEC Co., Ltd, China). The recording electrode of f-OCP was placed trans-nasally beneath the optic chiasm, and the reference electrode was placed at the temporal periorbital region of the testing eye. F-OCP shared the same ground electrode with f-VEP.

F-ONP recording was carried out with its recording electrode inserted into the chiasmatic ON. The reference and ground electrodes of f-ONP were the same as f-OCP.

### Light blocking and detector of region-biased retinal sensitivity

Traditional f-VEP used full-field light stimulation. To reduce light stimulation or achieve region-biased retinal stimulation, we used a handheld mini-Ganzfeld stimulator 12, and designed a custom-made light blocker (TAMIYA Inc., XF1, Japan), the curvature of which was the same as that of the stimulator, to conceal several quadrants of the light-emitting area of the stimulator (**Figure 2F, 5C**).

### Electrical stimulation and electrically evoked potential recording

This study used two kinds of electrical stimulations to establish an electrical field along the ON between the eye and the optic chiasm. The negative pole (cathode) was connected to the skull base BMI beneath the optic chiasm for the eye-to-chiasma electrical stimulation. The negative pole (cathode) was connected to the skull screw fastened to the occipital bone for the eye-to-occipital electrical stimulation. Both electrical stimulations shared the same positive pole (anode) (**Figure 5G**). The anode and cathode were connected to an electrical stimulator (MadLab-4C/5H, Beijing Zhongshidichuang Science and Technology Development Co., Ltd, China. Voltage of stimulation was set from 5-100 mV) to generate a series of electrical stimuli (positive voltage continuous rectangular wave) at the frequency of 1 Hz with a duration of 0.2 ms.

Electrodes were placed in the same way as f-OCP recording and electrically evoked potential between the eye and optic chiasm was recorded (**Figure 5G**).

### Pattern electroretinogram (p-ERG) and flash electroretinogram (f-ERG) recordings

P-ERG amplitude indicates the functional integrity of RGCs. P-ERG recording in goats has been previously reported 12. Briefly, the goat was anesthetized with xylazine. After electrode placement, p-ERG signals were elicited by contrast-reversal black-white checkerboards at different spatial frequencies (GT-2008V-Ⅲ, GOTEC Co., Ltd, China). F-ERG recording in goats was carried out using the same electrodes as p-ERG as described previously 26.

### Spectral-domain optical coherence tomography (SD-OCT) imaging

Retinal OCT imaging was used to measure the thickness of GCC including the RNFL, RGC, and IPL layers, representing RGC axons, somas, and dendrites, respectively. This method has been previously described 12. Briefly, after anesthesia with xylazine, retinal OCT images were taken using a peripapillary circular scan pattern (Heidelberg Spectralis OCT system, Germany). The GCC thickness was measured manually with the aid of the Heidelberg software.

### Fourier transform

(1) Description and equation:

The Fourier transform (FT) is a mathematical transform which decomposes function depending on temporal information (t) into function depending on temporal frequency (ω). Mathematical equation:



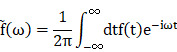



(2) Fast Fourier transform algorithm was used to transform the signal in time domain to the same signal in frequency domain. Source code in Matlab software (R2018b) was as follow:

*data_fft_output = fft(data_input);*

### Modulation transfer function (MTF) analysis

(1) Description:

The modulation transfer function (MTF) was defined as the magnitude response of the system (output signal) to different frequencies (input signal) which can obtained by calculating the ratio of the amplitude to the signals in frequency domain before and after the system.

(2) Mathematical equation and flowchart:

MTF at each light intensity was expressed as:



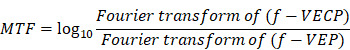



The amplitude of frequency spectrum was shown on natural logarithm scale. The flowchart is shown in **Figure S4.**

(3) Source code in Matlab software (R2018b):

*mtf = log10(abs(data_fft_1)/abs(data_fft_2)); % data_fft_1 and data_fft_2 are signal after Fourier transform.*

### Wavelet analysis

(1) Description and mathematical equation:

Wavelet analysis can be viewed as an alternative to Fourier transform, which decomposes function depending on time into function depending on both temporal frequency and time. In this research, we used a Morse wavelet to obtaining the time-frequency spectrum, which has been described in detail previously 27-29. General Fourier-domain form of the generalized Morse wavelets is:



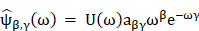



U(ω) is the unit step function, aβγ is the normalizing constant, ω is frequency, β is the order parameter, and γ is the symmetry or the family parameter.

(2) Source code in Matlab software (R2018b)

*[data_wavelet_output, fs, coi] = cwt(data_input, 'morse', frequency);*

The source code for the aforementioned three frequency domain analyses (FT, MTF, wavelet analysis) in Matlab software (R2018b) is as follows:

*% frequency domain analyses*

*fft_length = 500;*

*str_data_folder_1 = './01';*

*str_data_folder_2 = './02';*

files = dir([str_data_folder_1, '/*.txt']);

*time_gap = 250/500; % ms*

*time_gap_sescond = 250/500/1000; % s*

*frequency = 1/time_gap_sescond;*

xs = (1:fft_length) .* time_gap;

*data_ori = zeros(1, fft_length);*

xs_f = frequency *(0:(fft_length/2-1)) / fft_length;

*for idx = 1:length(files)*

*disp(['Process file:', files(idx).name]);*

*[~, file_name, ~] = fileparts(files(idx).name);*

*data = importdata([str_data_folder_1, '/', files(idx).name]);*

*data_ori_1 = [data, zeros(1, fft_length-length(data))];*

*data_fft_1 = fft(data_ori_1);*

*data = importdata([str_data_folder_2, '/', files(idx).name]);*

*data_ori_2 = [data, zeros(1, fft_length-length(data))];*

*data_fft_2 = fft(data_ori_2);*

*[data_wavelet_1, fs_1, coi] = cwt(data_ori_1, 'morse', frequency);*

*[data_wavelet_2, fs_2, coi] = cwt(data_ori_2, 'morse', frequency);*

*% save to figure*

*f = figure('units','inch','position',[0,0,15,10]);*

*subplot(4, 2, 1);*

*plot(xs, data_ori_1);*

*title('FVEP 01')*

*xlabel('time(ms)');*

*ylabel('Voltage(uv)');*

*subplot(4, 2, 2);*

*plot(xs, data_ori_2);*

*title('FVEP 02')*

*xlabel('time(ms)');*

*ylabel('Voltage(uv)');*

*subplot(4, 2, 3);*

*amp_log = log10(abs(data_fft_1));*

*plot(xs_f, amp_log(1:1:fft_length/2));*

*title('Amplitude 01');*

*subplot(4, 2, 4);*

*amp_log = log10(abs(data_fft_2));*

*plot(xs_f, amp_log(1:1:fft_length/2));*

*title('Amplitude 02');*

*subplot(4, 2, 5);*

*mesh(xs, fs_1, log10(abs(data_wavelet_1)));*

*view([142 13])*

*subplot(4, 2, 6);*

*mesh(xs, fs_2, log10(abs(data_wavelet_2)));*

*view([142 13])*

*subplot(4, 2, 7, 8);*

*mtf = log10(abs(data_fft_1)/abs(data_fft_2));*

*plot(xs_f, mtf(1:1:fft_length/2));*

*print(f, ['./', file_name, '.png'], '-dpng', '-r600');*

*close(f);*

*% write to file*

*csvwrite(['./', file_name, '_fft_1.csv'], data_fft_1);*

*csvwrite(['./', file_name, '_fft_2.csv'], data_fft_2);*

*csvwrite(['./', file_name, '_wavelet_1.csv'], data_wavelet_1);*

*csvwrite(['./', file_name, '_wavelet_2.csv'], data_wavelet_2);*

*end*

### Statistical analyses

All data were analyzed using GraphPad (9.0) software. Normality tests were used to analyze the distributions of all data sets. Student's t-test or nonparametric test was used to compare two groups of data. A one-way ANOVA (with Dunnett's multiple-comparisons test) or nonparametric Kruskal-Wallis test (with Dunn's multiple-comparisons test) was used to compare multiple groups. Two-way ANOVA (with multiple comparisons) was used to analyze OCT, p-ERG, f-VEP, and f-OCP. Asterisks (*) represent statistically significant differences (* p < 0.05, ** p < 0.01, *** p < 0.001, **** p < 0.0001). Data are presented as mean ± s.e.m. Statistical analysis method and detailed statistical data for each figure have been described and justified in the corresponding figure legend or **Table S1**.

### Data availability

All data generated or analyzed during this study are included in the manuscript and supporting files. We have uploaded a data file named “source data.zip”, including all numerical data for the figures in the manuscript.

## Discussion

The skull base of humans includes many essential neurovascular tissues and endocrine organs. However, few BMIs are implanted to the skull base due to its deep anatomic position. In this study, we confirmed the feasibility and safety of implantation of skull base BMI using minimally invasive trans-sphenoidal endoscopy. We further found that a skull base electrode placed beneath the optic chiasm recorded high amplitude f-OCP. Besides, f-OCP was significantly more sensitive to light stimulus and its changes than f-VEP, was more repeatable than f-VEP, and its signal disappeared when light stimulation was off, or the ON was transected completely, indicating f-OCP originated from ONP. We further demonstrated other applications of chiasmatic BMI and f-OCP, including the real-time detector of ON function, detector of region-biased retinal sensitivity, and safe therapeutic electrical stimulator with low excitation threshold.

### Potential applications of chiasmatic electrode

#### *In vivo* tracking function of RGC subtypes

RGC subtypes can be activated by different visual stimulations and have their specific electrophysiological signatures 30-32. Both Fourier transform and wavelet analysis of f-OCPs found that different light stimulations resulted in diverse frequency components (**Figure 5J-K**), probably generated from different output channels of RGC subtypes. In the future, we plan to capture OCP signatures of RGC subtypes by optogenetic methods 31, 33, and then try to track the function of RGC subtypes longitudinally.

### Deciphering visual processing

OCP was the input of the visual signal, while VEP was the output signal that was processed by the lateral geniculate nucleus and primary visual cortex. Comparison and analysis of f-OCP and f-VEP would help decipher the pattern of visual processing and visual prosthetics 34, 35. As a proof of concept, we performed modulation transfer function analysis between f-OCP and f-VEP and found that amplitudes at different frequencies were modulated by the visual processing system (**Figure 5L**).

### Visual prosthesis

ON is a potential target for the implantation of visual prosthesis 34-36. Although the diameter of ON is much smaller than the size of the retina or primary visual cortex, it provides access to the entire visual field. Previous studies found that cortical potential generated by electrical stimulation of ON had a wave shape similar to that generated by light stimulation 37, and visual sensation (phosphene) was elicited by electrical stimulation of ON 38-40. Traditional ON visual prosthesis was implanted at the intra-cranial, intra-orbital 41 or intraocular segment 38, where ON is surrounded by crowded tissues (e.g., the intra-orbital ON is surrounded by many vessels, nerves, muscles and adipose tissue 42). Our study demonstrated that ON visual prosthesis could be implanted microinvasively at the optic canal or optic chiasm within the spacious airy sphenoid sinus (**Figure 1E-G**).

### Broad applications of skull base BMIs

**Figure 6:**
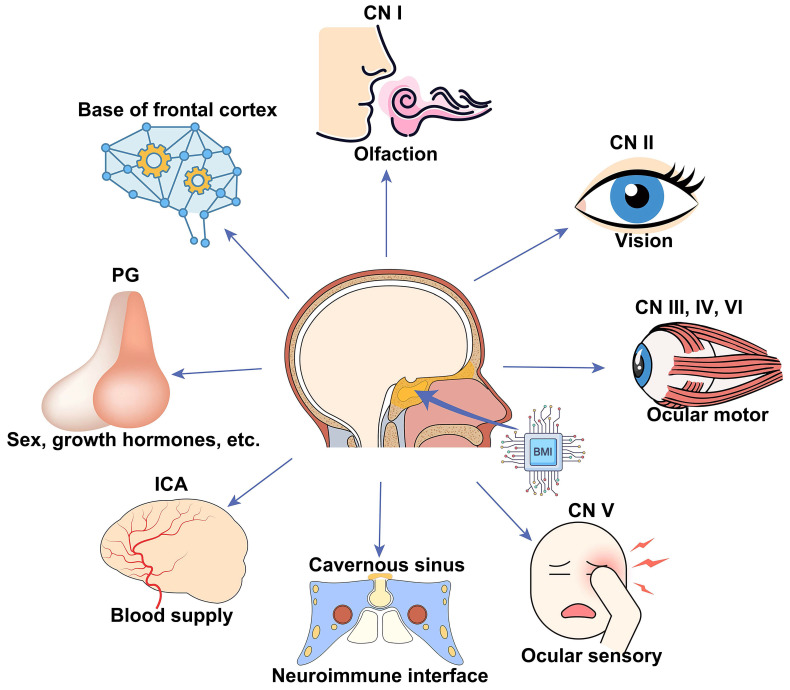
** Schematic of broad applications of skull base BMIs.** Potential applications, including but not limited to, monitoring and modulating activities of the bottom of the frontal cortex, cranial nerve (CN I-VI), internal carotid artery (ICA) and cavernous sinus, and pituitary gland.

BMIs implanted at the skull base via minimally invasive trans-sphenoidal endoscopy have broad applications in monitoring and modulating activities of the following important neurovascular tissues lying over the skull base (**Figure 6**), including the frontal lobe 43, half of the CNs (CN I to VI), ICA 44, cavernous sinus 45, and the pituitary gland. For example, CN I (olfactory nerve) is in charge of the sense of smell, which became dysfunctional in patients with COVID-19 46 and long-duration astronauts 47. Electrical stimulation to the skull base electrode placed on the CN I via the trans-nasal microinvasive approach may help explore the underlying electrophysiological mechanism of olfactory function and restore olfactory function by inducing artificial sense of smell 48, 49.

### Animal species for testing skull base BMIs

Here we employed goats as an animal model, due to the similarities to humans in size and anatomy of the nasal cavity, to implant skull base BMI trans-nasally. Additionally, goats are relatively cheap and available compared to non-human primates. Previously, we had successfully exposed the skull base in the rhesus macaque, minipig, and beagle using the same trans-sphenoidal endoscopy (data unpublished). Therefore, trans-sphenoidal skull base BMIs can be tried and optimized in the goat and then tested in rhesus macaques before clinical application.

## Limitations

Although the current transplantation of chiasmatic electrode did not cause detected abnormality in fetching/moving behaviors, skull/orbital CT scans, and ophthalmic tests over months after surgery, it required trans-sphenoidal endoscopy to partially remove the middle and superior turbinates as well as the anterior wall of the sphenoid sinus to exposure the sphenoid sinus. Therefore, the current direct clinical application of this microinvasive method may limit to patients who need trans-sphenoidal endoscopy for treatment purposes. In the near future, we will work on developing smaller and wireless chiasmatic electrode, which can be transplanted through the sphenoid ostium (**Figure 1E**), the natural opening of the sphenoid sinus, without the need of surgical removal of the turbinates and the anterior wall of the sphenoid sinus. Since the same chiasmatic electrode can be potentially used for therapeutic electrical stimulation of the injured optic nerve (**Figure 5G-I**), its clinical applicability might expand to patients with optic neuropathy for monitoring and treatment purposes.

Additionally, the current chiasmatic electrode is relatively too large for rodents, limiting its application in small animal studies.

We understand this may not be a typical BMI study involving advanced bio-material, circuit or algorithm. However, methodological advances in BMI should also include safe surgical approaches for implantation and model organisms for the pre-clinical test to achieve widespread clinical adoption of BMI. We believe that our work will generate the broad interest of basic scientists, engineers, and clinicians in the fields of BMIs, neuroscience, cerebral vasculature, and endocrine functions. Furthermore, we hope our work would encourage more surgeons to take an active role in BMI progress 50.

## Conclusions

Our study established a novel diagnostic method of OCP to evaluate optic nerve function in a clinically-relevant large animal model (goat) using a microinvasive skull base electrode at the optic chiasm. Compared with currently used VEP, the OCP signal is an order of magnitude stronger, more sensitive, and stable. Another important feature of the procedure is that it is swift and safe. Thus, our study provides a new route for skull base brain-machine interface with minimal surgical invasion. This method will be valuable for animal models studying visual function and outcome measurement of treatments. More significantly, our procedure is applicable to clinical cases with traumatic visual defects.

## Supplementary Material

Supplementary figures and table.

Supplementary movie 1.

Supplementary movie 2.

Supplementary movie 3.

## Abbreviations

VEP: visual evoked potential
ONP: optic nerve potential
OCP: optic chiasmatic potential
BMI: Brain-machine interface
CN: cranial nerves
ICA: intra-carotid artery
SS: sphenoid sinus
EB: ethmoid bulla
ST: superior turbinate
SO: sphenoid ostium
CT: Computed Tomography
OCT: optic coherent tomography
GCC: ganglion cell complex
p-ERG: pattern electroretinogram
RGCs: retinal ganglion cells
CV: coefficient of variation
mpi: month post-injury
FT: Fourier transform
MTF: Modulation transfer function
