# Supplementary material for: Optic chiasmatic potential by endoscopically implanted skull base microinvasive biosensor: a brain-machine interface approach for anterior visual pathway assessment
Source: Theranostics. 2022 Apr 11;12(7):3273–87. doi: 10.7150/thno.71164 (PMC9065198; doi:10.7150/thno.71164)
Supplement: Supplementary file 1 — Supplementary figures and table. [file thnov12p3273s1.pdf]

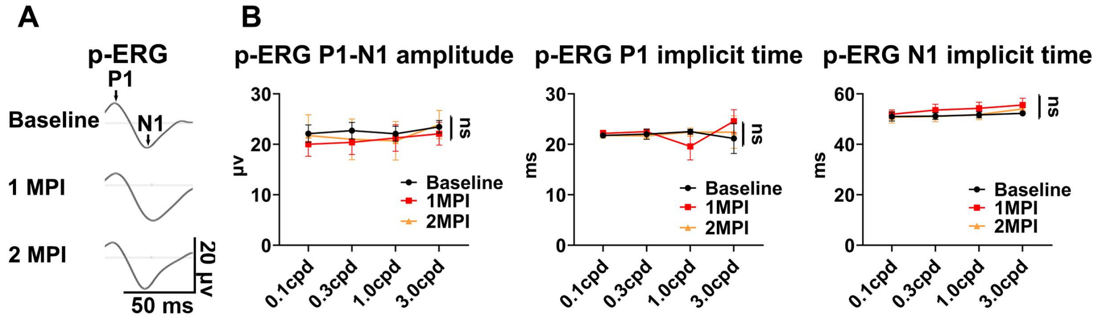

Supplementary figure S1. Skull base BMI implantation does not cause loss of visual function. (A) Representative waveforms of p-ERG before and after skull base BMI implantation. (B) Quantification of amplitudes and implicit times of p-ERG. N = 6 goats, two-way ANOVA with Dunnett's multiple comparisons. Data are presented as mean ± s.e.m, ns: not significant.

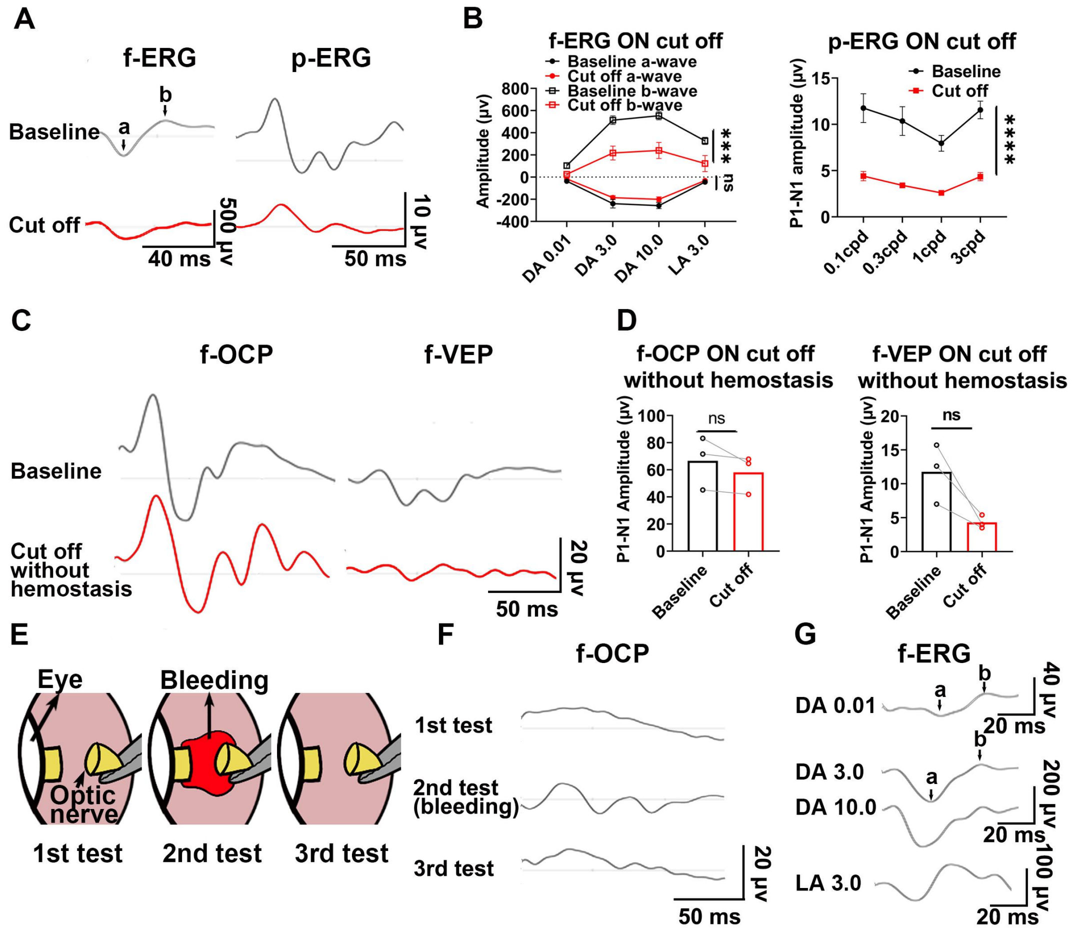

Supplementary figure S2. (A) Representative waveforms of f-ERG and p-ERG before and after ON cutoff. (B) Quantification of a, b-wave amplitude of f-ERG and P1-N1 amplitude of p-ERG before and after ON cutoff. N = 6 goats for f-ERG, two-way ANOVA with Tukey's multiple comparisons. N = 2 goats for p-ERG, two-way ANOVA. Data are presented as mean ± s.e.m. (C) Representative waveforms of f-OCP and f-VEP before and after ON cutoff without hemostasis. (D) Quantification of f-OCP and f-VEP P1-N1 amplitudes in (C). N = 3 goats, paired t-test. (E) Schematic of three consecutive f-OCP tests with or without complete hemostasis. (F) Three f-OCP waveforms in (E). (G) FERG waveforms recorded immediately after 3rd f-OCP test.Ns: not significant, **: $p < 0.01$, ****: $p < 0.0001$.

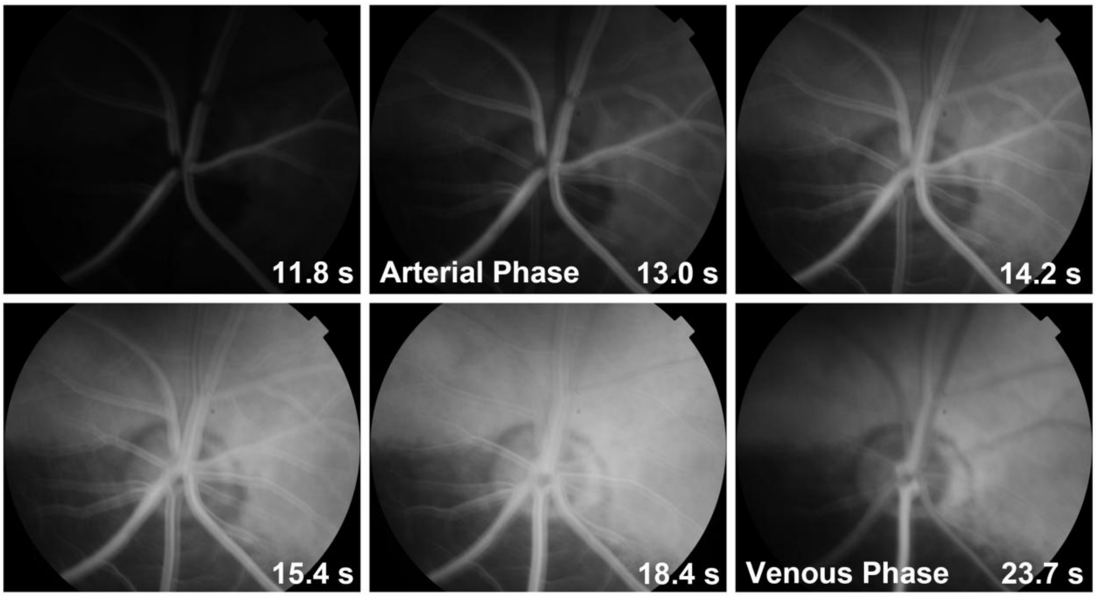

Supplementary figure S3. Fundus fluorescein angiography (FFA) shows normal retinal vascular irritation after careful retrobulbar ON transection in the goat. A consecutive series of FFA images are shown. The periods from ear-vein injection of fluorescein sodium to the complete filling of the retinal arterial and venous vessels are defined as the arterial phase time and venous phase time respectively.

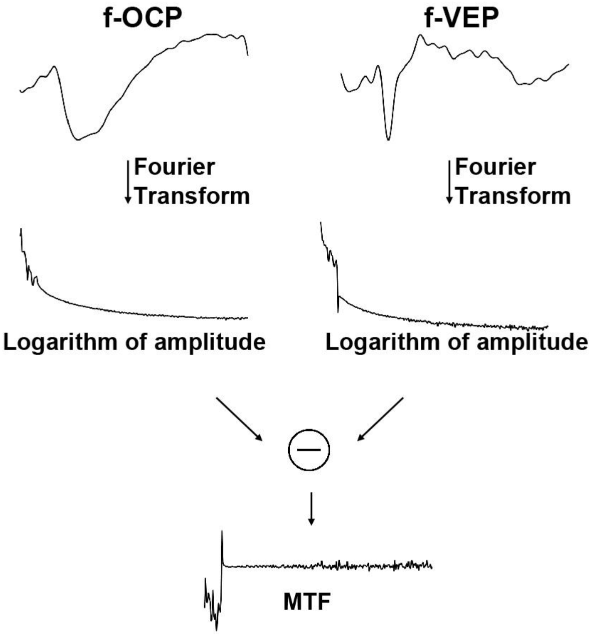

Supplementary figure S4. The flowchart of MTF analyse.

Supplementary movies 1: Normal behaviors after trans-nasal implantation of chiasmatic electrode via minimally invasive endoscopy

Supplementary movies 2: Skull CT scans 1 day after trans-nasal implantation of the chiasmatic electrode via minimally invasive endoscopy

Supplementary movies 3: Skull CT scans 3 months after trans-nasal implantation of the chiasmatic electrode via minimally invasive endoscopy

Supplementary table: Statistical details for each figure.

	**Statistical method**	**One/two-tailed**	**n values**	**P values**	**F values/t values**	**degrees of freedom**
Figure 1M	Two-way ANOVA with Dunnett's multiple comparisons test	two-tailed	n=6	P=0.9954, 0.9827 between baseline and 1mpi, 2mpi	/	DF=100
Figure 2C	Two-way ANOVA with Šídák's multiple comparisons test	two-tailed	n=4 (no light stimulation), n=7 (other light intensities)	P=>0.9999, 0.0042, <0.0001, <0.0001, <0.0001 between f-OCP and f-VEP under 0, 0.005, 0.025, 0.05, 0.25 cd·s/m^2	t=0.09071, 3.538, 5.826, 7.090, 8.931 under 0, 0.005, 0.025, 0.05, 0.25 cd·s/m^2	DF=54
Figure 2D	Two-way ANOVA with Tukey's multiple comparisons test	two-tailed	n=7	P=0.0282 between f-OCP and f-VEP at 5x, 10x, 50x	/	DF=38

Figure 2D	Friedman test with Dunn's multiple comparisons test	two-tailed	n=7	P=0.2931, 0.0113, 0.0001 between 1x and 5x, 10x, 50x in f-OCP<br>P=>0.9999, 0.7646, 0.7646 between 1x and 5x, 10x, 50x in f-VEP	/	/
Figure 2E	Two-way ANOVA	two-tailed	n=4	P=0.9377, <0.0001 between f-OCP and f-VEP in P1, N1 implicit times	F=0.006223, 24.50 in P1, N1 implicit times	DF=1
Figure 2G	RM one-way ANOVA with Dunnett's multiple comparisons test	two-tailed	n=6	P=0.4072, 0.0184, <0.0001 between no light and 3/4LB, 1/2LB, no LB in f-OCP;<br>P=0.8777, 0.1561, 0.0014 between no light and 3/4LB, 1/2LB, no LB in f-VEP	/	DF=15
Figure 2H	Paired t test	two-tailed	n=5	P=0.0011, 0.0446 between f-OCP and f-VEP in reference and recording electrodes	t=8.329, 2.889in reference and recording electrodes	DF=4
Figure 3B	Paired t test	two-tailed	n=14	P=0.0013	t=4.068	DF=13

Figure 3D	Two-way ANOVA with Tukey's multiple comparisons test	two-tailed	n=11	P=<0.0001 between f-OCP and f-VEP under 0, 0.005, 0.025, 0.05, 0.25 cd·s/㎡	/	DF=62
Figure 3F	Two-way ANOVA	two-tailed	n=11	P=0.0153 between f-OCP and f-VEP	F=6.227	DF=1
Figure 3G	Two-way ANOVA with Tukey's multiple comparisons test	two-tailed	n=11	P=<0.0001 between f-OCP and f-VEP under 0, 0.005, 0.025, 0.05, 0.25 cd·s/㎡	/	DF=62
Figure 4C	RM one-way ANOVA with Dunnett's multiple comparisons test	two-tailed	n=3	P=0.0625, 0.0457 between 0(IPS) and 0.25(CL), 0.25(IPS) cd·s/㎡	/	DF=2
Figure 4F left panel	Two-way ANOVA	two-tailed	n=3	P=0.0199	F=6.899	DF=1
Figure 4F middle panel	Two-way ANOVA	two-tailed	n=3	P=0.8403	F=0.04212	DF=1
Figure 4F right panel	Two-way ANOVA	two-tailed	n=3	P=0.7265	F=0.1274	DF=1
Figure 4I left panel	Ratio paired t test	two-tailed	n=3	P=0.0341	t=5.275	DF=2
Figure 4I right panel	Mann Whitney test	two-tailed	n=3	P=0.1000	/	/

Figure 4L	RM one-way ANOVA with Dunnett’s multiple comparisons test	two-tailed	n=3	P=0.9510, 0.6319, 0.9723, 0.5799 between mid and left, right, up, down	/	DF=2
Figure 5B	Two-way ANOVA	two-tailed	n=3	P=0.0309	F=5.758	DF=1
Figure 5E	Two-way ANOVA with Tukey's multiple comparisons test	two-tailed	n=3	P=0.0035 between eye-to-chiasm and eye-to-occipital; P=0.0333 between eye-to-chiasm and eye-to-occipital under 5, 50, 500mv	F=12.29 for two-way ANOVA	DF=1 for two-way ANOVA; DF=14 for Tukey’s multiple comparisons test
Figure S1B left panel	Two-way ANOVA with Dunnett's multiple comparisons test	two-tailed	n=6	P=0.5690, 0.8812 between baseline and 1mpi, 2mpi	/	DF=66
Figure S1B middle panel	Two-way ANOVA with Dunnett's multiple comparisons test	two-tailed	n=6	P=0.9372, 0.9777 between baseline and 1mpi, 2mpi	/	DF=66
Figure S1B right panel	Two-way ANOVA with Dunnett's multiple comparisons test	two-tailed	n=6	P=0.1389, 0.9405 between baseline and 1mpi, 2mpi	/	DF=66

Figure S2B left panel	Two-way ANOVA with Tukey's multiple comparisons test	two-tailed	n=4	P=0.8864 between Baseline a-wave and Cut off a-wave; P=0.0002 between Baseline b-wave and Cut off b-wave	/	DF=57
Figure S2B right panel	Two-way ANOVA	two-tailed	n=2	P=<0.0001 between Baseline and Cut off	F=120.3	DF=1
Figure S2D left panel	Paired t test	two-tailed	n=3	P=0.2345	t=1.682	DF=2
Figure S2D right panel	Paired t test	two-tailed	n=3	P=0.0878	t=3.149	DF=2
